# Clinical outcomes of intrauterine device insertions by newly trained providers: The ECHO trial experience

**DOI:** 10.1016/j.conx.2023.100092

**Published:** 2023-03-30

**Authors:** Irina Yacobson, Valentine Wanga, Khatija Ahmed, Tsungai Chipato, Peter Gichangi, James Kiarie, Cheryl Louw, Susan Morrison, Margaret Moss, Nelly R. Mugo, Thesla Palanee-Phillips, Melanie Pleaner, Caitlin W. Scoville, Katherine K. Thomas, Kavita Nanda, for the Evidence for Contraceptive Options and HIV Outcomes (ECHO) Trial Consortium

**Affiliations:** a FHI 360, Durham, NC, United States; b Department of Global Health, University of Washington, Seattle, WA, United States; c Setshaba Research Centre, Soshanguve, Gauteng, South Africa; d Faculty of Health Sciences, Department of Medical Microbiology, University of Pretoria, Pretoria, Gauteng, South Africa; e University of Zimbabwe, College of Health Sciences, Harare, Zimbabwe; f International Centre for Reproductive Health, Nyali, Mombasa, Kenya; g University of Nairobi, Nairobi, Kenya; h Department of Public Health and Primary Care, Faculty of Medicine and Health Sciences, Ghent University, Ghent, East Flanders, Belgium; i UNDP-UNFPA-UNICEF-WHO-World Bank Special Programme of Research, Development and Research Training in Human Reproduction, World Health Organization, Geneva, Switzerland; j Madibeng Centre for Research, Brits, North West, South Africa; k Department of Family Medicine, University of Pretoria, Pretoria, Gauteng, South Africa; l Department of O&G, Faculty of Health Sciences, University of Cape Town/Groote Schuur Hospital, Cape Town, Western Cape, South Africa; m Center for Clinical Research, Kenya Medical Research Institute, Nairobi, Kenya; n Wits Reproductive Health and HIV Institute, University of the Witwatersrand, Johannesburg, Gauteng, South Africa

**Keywords:** Copper intrauterine device, Expulsion, Insertion, IUD, Provider training, Uterine perforation

## Abstract

**Objectives:**

To assess the rates of failed insertion, expulsion, and perforation when intrauterine device (IUD) insertions were done by newly trained clinicians, and to examine factors that may affect these outcomes.

**Study design:**

We evaluated skill-based outcomes following IUD insertion at 12 African sites in a secondary analysis of the Evidence for Contraceptive Options and HIV Outcomes (ECHO) randomized trial. Before trial initiation, we provided competency-based IUD training to clinicians and offered ongoing clinical support. We used Cox proportional hazards regression to examine factors associated with expulsion.

**Results:**

Among 2582 IUD acceptors who underwent first attempted IUD insertion, 141 experienced insertion failure (5.46%) and seven had uterine perforation (0.27%). Perforation was more common among breastfeeding women within three months postpartum (0.65%) compared with non-breastfeeding women (0.22%). We recorded 493 expulsions (15.5 per 100 person-years, 95% confidence interval [CI] 14.1─16.9): 383 partial and 110 complete. The risk of IUD expulsion was lower among women older than 24 years (aHR 0.63, 95% CI 0.50─0.78) and may be higher among nulliparous women. (aHR 1.65, 95% CI 0.97─2.82). Breastfeeding (aHR 0.94, 95% CI 0.72─1.22) had no significant effect on expulsion. IUD expulsion rate was highest during the first three months of the trial.

**Conclusions:**

IUD insertion failure and uterine perforation rates in our study were comparable to those reported in the literature. These results suggest that training, ongoing support, and opportunities to apply new skills were effective in ensuring good clinical outcomes for women receiving IUD insertion by newly trained providers.

**Implications:**

Data from this study support recommendations to program managers, policymakers, and clinicians that IUDs can be inserted safely in resource-constrained settings when providers receive appropriate training and support.

## 1 Introduction

The copper intrauterine device (IUD) is among the most effective and widely used contraceptive methods worldwide [1]. Globally, 14.3% of reproductive-age women rely on intrauterine contraception. However, global distribution is highly uneven, with>80% of world IUD users living in Asia but only 0.5% in Sub-Saharan Africa [2]. Low IUD utilization means fewer opportunities for providers to acquire and maintain IUD insertion skills. This is particularly important for recently trained providers who may lose confidence without opportunities to regularly apply new skills. Adverse clinical outcomes associated with IUD use, such as uterine perforation and IUD expulsion, are linked to provider experience [3], [4], [5].

The Evidence for Contraceptive Options and HIV Outcomes (ECHO) trial (December 2015–October 2018) was a multicenter randomized clinical trial comparing the risk of HIV acquisition among users of three contraceptive methods—intramuscular depot medroxyprogesterone acetate, a levonorgestrel implant (Jadelle), and a T-380-A copper IUD. The trial also compared side effects, pregnancy rates, and women’s patterns of contraceptive use [6]. Most ECHO clinicians had either limited or no experience with providing IUDs and were trained before trial initiation. ECHO offered a unique opportunity to assess rates of skill-based outcomes, such as failed insertions, expulsions, and uterine perforations, when IUD insertions were done by recently trained clinicians, and to examine factors that may affect such outcomes.

## 2 Material and methods

Between December 2015 and September 2017, the ECHO trial enrolled 7829 women at 12 research sites in four African countries (nine sites in South Africa, and one site each in Kenya, Eswatini, and Zambia) selected for high HIV incidence and geographical distribution. All women were sexually active, aged 16 to 35 years, desired effective contraception for at least 18 month, and consented to be randomized to any of the three contraceptive methods. At enrollment, study participants were randomly assigned (1:1:1) to levonorgestrel implant (*n* = 2613), intramuscular depot medroxyprogesterone acetate (*n* = 2609), or copper IUD (*n* = 2607) and had follow-up visits scheduled at 1 month post-enrollment and then every 3 month up to a maximum of 18 month. Participants were retained in the trial even if they discontinued their randomized method, which they could choose to do at any point. Trial methods and results were described previously [6]. The trial was approved by institutional review boards at participating sites and all participants provided written informed consent.

We conducted a secondary analysis of ECHO trial data to (1) assess rates of IUD expulsion, perforation, and insertion failure; (2) evaluate demographic risk factors for IUD expulsion; and (3) explore the association between expulsion and months since the start of enrollment at each study site as a proxy for provider experience with IUD insertions. We did not include pelvic inflammatory disease in this analysis because post-insertion infectious complications are linked mainly to the presence of undiagnosed infection at the time of insertion and are being addressed in a separate manuscript.

### 2.1 Provider training

Before trial initiation, we conducted 5-day competency-based training workshops for 54 ECHO research clinicians (doctors and nurses) who were expected to insert IUDs over the course of the trial. The theoretical portion of the training covered technical information on the trial contraceptive methods and contraceptive counseling; a skill-building component included a full day practicing IUD insertion technique using anatomical models.

After this initial training, we required each clinician to complete 10 proficient, independent IUD insertions in a regular family planning setting under the supervision of an ECHO clinical trainer who used a standardized checklist for grading.^1^ We defined proficient insertions as those where all tasks were performed correctly and in proper sequence. Upon completion of 10 proficient insertions, we considered a clinician certified to insert IUDs in trial participants.

### 2.2 Additional clinical support and quality assurance

We required each trial site to have an independent backup gynecologist with IUD experience to be available for failed insertions, suspected perforations, or for confirmation of suspected complete expulsions. If IUD insertion failed on the first attempt, we recommended that subsequent attempts be made by the backup gynecologist. We also had two ECHO contraceptive experts based in North Carolina, USA, who reviewed detailed reports of all IUD-related adverse outcomes within 24 hours to ensure appropriate management and follow-up. Additionally, ECHO contraceptive experts facilitated monthly conference calls with trial clinicians to provide refreshers on selected clinical management issues and share successes and challenges across trial sites.

Two ECHO clinical trainers and an ECHO counseling expert conducted on-site clinical support and quality assurance visits approximately halfway through the trial period with additional visits at sites with more frequent expulsions or other concerns.

### 2.3 Definitions and IUD-specific trial procedures

We defined insertion failure as an attempted insertion that did not result in successful IUD placement. We defined expulsions as partial when the IUD stem protruded from the external cervical os on a pelvic exam or was noted below the internal cervical os by ultrasound. We defined expulsions as complete when a woman reported not being on IUD continuously since her last study visit (i.e., saw IUD being expelled), and/or IUD absence was confirmed during a pelvic examination and/or imaging at the current visit.

We assigned the dates of expulsions as follows: for partial expulsions, the date when it was first diagnosed on pelvic examination or ultrasound; for complete expulsions that occurred without a woman noticing, we assigned the expulsion date as the midpoint between the last time IUD presence was verified at a study visit and the date the expulsion was discovered by the trial clinician. In cases of pregnancy where the IUD was not seen on pelvic examination, ultrasound, or at the time of delivery, we assumed that IUD expulsion occurred before pregnancy and assigned the date of expulsion as the midpoint between last IUD verification date and the estimated date of fertilization.

At the 1 month and final visits, clinicians confirmed IUD presence by speculum exam, and during all quarterly follow-up visits, asked if women had any complaints and if they were still using the IUD. Clinicians conducted pelvic examinations during follow-up visits when expulsion, perforation, or infection were suspected based on symptoms and signs, such as intermenstrual cramps, irregular vaginal bleeding/spotting, pain during intercourse, abnormal vaginal discharge, or lower abdominal pain. They referred women to the backup gynecologist to confirm all suspected complete expulsion or uterine perforations by ultrasound or X-ray, and to evaluate for partial expulsion by ultrasound when IUD strings appeared longer than expected. Women with diagnosed expulsion were offered a new IUD inserted during the same visit.

### 2.4 Statistical analysis

We calculated IUD insertion failure and perforation rates following the initial IUD insertion attempt among all women with at least one attempt during the trial. For perforation, we also stratified data by breastfeeding status and time postpartum (i.e., less or more than 3 month).

We calculated incidence rates for complete, partial, and any expulsion among all women who had a successful IUD placement during the trial. We computed time at risk starting from the day IUD was inserted and stopping at the time the IUD was discontinued or expelled, dropping periods when a woman was not using the IUD from the analysis. Women reentered time at risk if they reinitiated the IUD; thus, some had recurrent outcomes. For a visual illustration, we plotted Kaplan-Meier estimates of time to the first expulsion. We used Cox proportional hazards regression with Anderson-Gill extension for multiple expulsions per woman to estimate associations between any expulsion and women’s age, parity, body mass index (BMI), breastfeeding status (all at baseline), and months since the start of enrollment for woman’s site (a proxy for clinician experience, time-varying); adjusted models included all the risk factors. We considered associations statistically significant when the 95% confidence interval (CI) of the estimated hazard ratio (HR) did not include 1.0.

### 2.5 Clinical trial registration

ECHO trial is registered with ClinicalTrials.gov, number NCT02550067.

## 3 Results

Among 2607 women randomized to IUD, 2582 (99%) accepted the IUD and underwent an IUD insertion attempt, and 2528 (97%) received the IUD. Among those attempting IUD insertion, the median age was 23 years; most (79.5%) were parous with a median time of 19 month since the end of the last pregnancy; nearly one-third (30.2%) were breastfeeding at the time of trial enrollment; and approximately half were either overweight (26.0%) or obese (25.4%) (Table 1).

**Table 1:** Baseline characteristics of women who had at least one intrauterine device placement attempt in the ECHO trial (*N* = 2582)

Characteristic	*N* (%) or median [IQR]
Age (y)	23 [20, 26]
16–17	26 (1.0%)
18–20	677 (26.2%)
21–24	901 (34.9%)
25–30	748 (29.0%)
31–35	230 (8.9%)
BMI (kg/m^2^)^a^	25 [22, 30]
<8.5 (Underweight)	116 (4.5%)
18.5–24.9 (Normal weight)	1138 (44.1%)
25–29.9 (Overweight)	671 (26.0%)
≥30 (Obese)	656 (25.4%)
Parous^b^	2052 (79.5%)
Months since last pregnancy ended	19 [6, 41]
Currently breastfeeding	781 (30.2%)

BMI, body mass index; ECHO, Evidence for Contraceptive Options and HIV Outcomes; IQR, Interquartile range; N (%), number (percent).

a One participant was missing BMI data.

b Participant has been pregnant at least once with no fetal loss before week 20; nulliparous otherwise.

### 3.1 IUD insertion failures

**Fig. 1:**
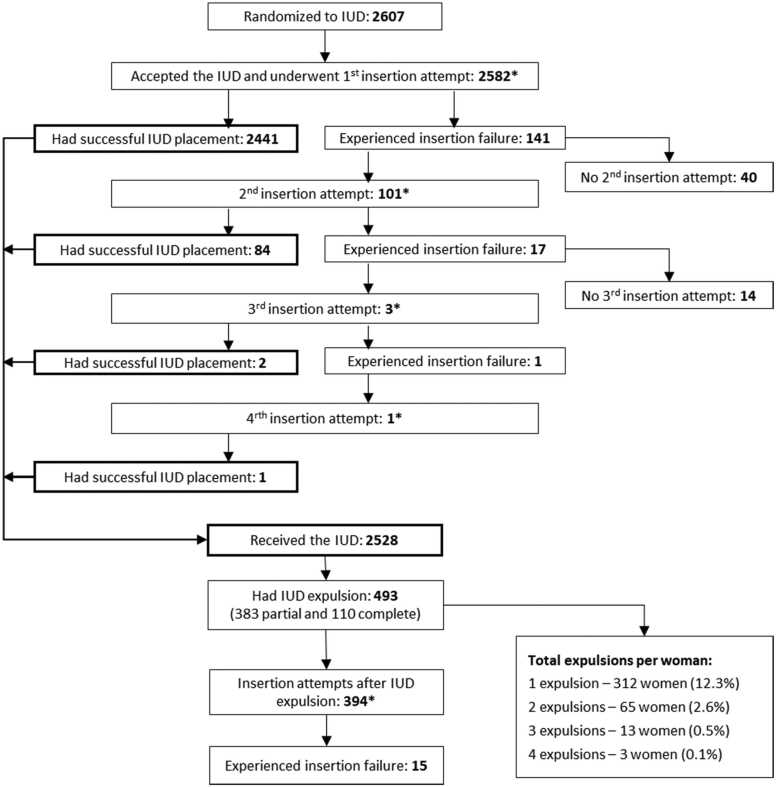
Summary of the IUD insertion attempts, failures, and expulsions over the course of the ECHO trial. *Contributed to the total number of IUD insertion attempts over the course of the trial (*N* = 3081). IUD = intrauterine device.

We recorded 141 IUD insertion failures (5.46%) among 2582 IUD acceptors during the first insertion attempt (Table 2). Another 18 failures occurred during the second and third insertion attempts following the initial insertion failure. Fifteen additional insertion failures occurred during 394 insertion attempts after IUD expulsions, leading to a total of 174 (5.64%) insertion failures among all 3081 IUD insertion attempts over the course of the trial (Fig. 1).

**Table 2:** Insertion failure and perforation following initial intrauterine device placement attempt in the ECHO trial

Outcome	Number with outcome/Number at risk	Percent
IUD insertion failure	141/2582	5.46
Perforation		
All women	7/2582	0.27
Non-breastfeeding women	4/1801	0.22
Breastfeeding women		
<3 month postpartum	2/306	0.65
≥3 month postpartum	1/475	0.21

ECHO, Evidence for Contraceptive Options and HIV Outcomes; IUD, intrauterine device.

### 3.2 Uterine perforations

We diagnosed seven uterine perforations (0.27%) among 2582 first attempted insertions (Table 2). No perforations occurred in women who reinitiated IUD after expulsion. Women who experienced perforation were 22 to 27 years of age and all were parous; three were breastfeeding at the time of perforation with two (0.65%) being within 3-month postpartum and one (0.21%) more than 3-month postpartum. Clinicians’ awareness of perforations varied significantly—from immediately at the time of insertion to 465 days later (Table 3). All but two perforations likely occurred at the time of insertion, though the diagnosis may have been confirmed by imaging days later. Three perforations were complete: one was recognized during uterine sounding, at which point the procedure was stopped and IUD insertion delayed; in two other cases the IUD was identified outside of the uterine cavity. Four perforations were partial with most of the IUD remaining in the uterine cavity in each case.

**Table 3:** Characteristics of women diagnosed with uterine perforation in the ECHO trial

Type of perforation	Awareness of perforation (days since insertion attempt)	Characteristics (at enrollment)		
		Age (in years)	Breastfeeding	Months since last pregnancy ended
Complete; by uterine sound (IUD insertion delayed)	0	25	No	16.9
Complete	0	26	Yes	17.8
Complete	39	27	Yes	2.8
Partial	465	25	No	14.6
Partial	66	27	No	27.6
Partial	28	24	No	65.6
Partial	7	22	Yes	2.3

ECHO, Evidence for Contraceptive Options and HIV Outcomes; IUD , intrauterine device.

### 3.3 IUD expulsions

**Fig. 2:**
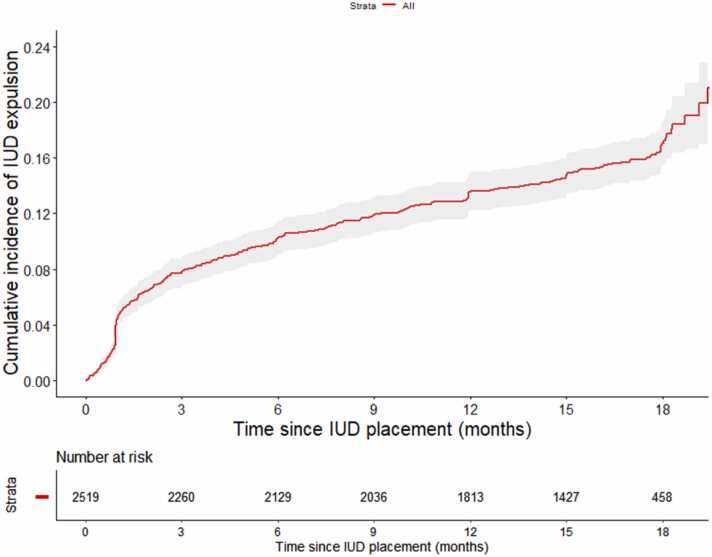
Cumulative incidence of the intrauterine device expulsion (time to first expulsion; 95% CI). CI = confidence interval; IUD = intrauterine device.

Overall, 493 expulsions (15.5/100 person-years, 95% CI 14.1–16.9) occurred among 2528 IUD users (Table 4): 383 partial (12.0/100 person-years, 95% CI 10.8–13.3) and 110 complete (3.5/100 person-years, 95% CI 2.8–4.2). The number of expulsions per woman ranged from one to four, with 81 women having more than one expulsion (Fig. 1). The cumulative incidence of a first IUD expulsion at months 6, 12, and 18 was 10.3%, 13.6%, and 17.0%, respectively (Fig. 2).

**Table 4:** Intrauterine device expulsion incidence rates among women who received it in the ECHO trial

Outcome	Number of women	Number of expulsions	Number of person-years at risk^a^	Incidence per 100 person-years (95% confidence interval)
Any expulsion	2528	493	3187	15.5 (14.1–16.9)
Partial expulsion	2528	383	3187	12.0 (10.8–13.3)
Complete expulsion	2528	110	3187	3.5 (2.8–4.2)

ECHO, Evidence for Contraceptive Options and HIV Outcomes; IUD , intrauterine device.

a The number of person-years at risk was calculated using days from IUD insertion to IUD expulsion or end of the study. If an expulsion was experienced during the study and the IUD was reinserted, days from expulsion to reinsertion do not contribute. However, after reinsertion, the woman is again considered at risk until the next IUD expulsion or end of the study.

Out of 493 expulsions, 45 were discovered during the final visit pelvic examination. Only 5 of these 45 women had symptoms suggestive of expulsion: 3 (6.7%) reported abdominal discomfort or pain, and 2 (4.4%) reported irregular bleeding or spotting at their final visit.

After adjusting for parity, BMI, breastfeeding status, and months since the start of enrollment at the study site, the risk of IUD expulsion was lower among women older than 24 years (adjusted HR [aHR] 0.63, 95% CI 0.50–0.78) and possibly higher among nulliparous women (aHR 1.65, 95% CI 0.97–2.82). Risk of expulsion was not significantly associated with breastfeeding (aHR 0.94, 95% CI 0.72–1.22), being overweight (aHR 1.11, 95 CI 0.87–1.41), or obese (aHR 1.08, 95 CI 0.83–1.41). The expulsion rate was highest during the first 3 month following the initial provider training (21.2/100 person-years, 95% CI 16.8–26.4) (Table 5).

**Table 5:** ECHO trial participant characteristics associated with the intrauterine device expulsion

Baseline characteristic	*N* women at risk	*N* expulsions	*N* person-years at risk^a^	Incidence per 100 person-years (95% CI)	Unadjusted HR (95% CI)	Adjusted^b^ HR (95% CI)
Age						
≤24 y	1561	351	1954	18.0 (16.1, 19.9)	Reference	Reference
>24 y	960	142	1236	11.5 (9.7, 13.5)	0.66 (0.54, 0.80)	0.63 (0.50, 0.78)
Parity						
Parous	2021	390	2587	15.1 (13.6, 16.7)	Reference	Reference
Nulliparous	500	103	603	17.1 (13.9, 20.7)	1.19 (0.95, 1.50)	1.65 (0.97, 2.82)
BMI (kg/m^2^)						
Normal weight	1113	222	1412	15.7 (13.7, 17.9)	Reference	Reference
Underweight	108	21	139	15.1 (9.4, 23.1)	0.98 (0.62, 1.54)	0.94 (0.56, 1.58)
Overweight	659	138	822	16.8 (14.1, 19.8)	1.07 (0.86, 1.33)	1.11 (0.87, 1.41)
Obese	640	112	816	13.7 (11.3, 16.5)	0.93 (0.73, 1.17)	1.08 (0.83, 1.41)
Breastfeeding						
No	1296	247	1644	15.0 (13.2, 17.0)	Reference	Reference
Yes	773	158	998	15.8 (13.5, 18.5)	0.94 (0.73, 1.22)	0.94 (0.72, 1.22)
						
Time varying characteristic	*N* women at risk (N observations)	*N* expulsions	*N* person-years at risk^a^	Incidence per 100 person-years (95% CI)	Unadjusted HR (95% CI)	Adjusted*** HR (95% CI)
Months since start of enrollment for participant’s site						
0–3	259 (325)	79	372	21.2 (16.8, 26.4)	Reference	Reference
3–6	461 (520)	84	639	13.1 (10.5, 16.3)	0.66 (0.48, 0.90)	0.63 (0.44, 0.89)
6–9	510 (578)	97	706	13.7 (11.1, 16.8)	0.68 (0.50, 0.92)	0.74 (0.53, 1.03)
9–12	470 (535)	85	617	13.8 (11.0, 17.0)	0.65 (0.47, 0.89)	0.66 (0.47, 0.94)
12+	821 (918)	148	856	17.3 (14.6, 20.3)	0.84 (0.63, 1.12)	0.84 (0.60, 1.16)

BMI, body mass index; CI, confidence interval; ECHO, Evidence for Contraceptive Options and HIV Outcomes; HR, hazard ratio; N, number; IUD, intrauterine device.

a Time at risk was calculated using days from IUD insertion to IUD expulsion or end of the study. If an expulsion was experienced during the study and the IUD was reinserted, days from expulsion to reinsertion do not contribute. However, after reinsertion, the woman is again considered at risk until the next IUD expulsion or end of the study.

b HRs from a model adjusted for age, parity, BMI, breastfeeding status, and months since the start of enrollment for the participant’s site.

## 4 Discussion

The ECHO trial offered a unique opportunity to assess the outcomes of IUD insertions conducted by recently trained clinicians. We only considered outcomes linked to provider clinical skills, such as failed insertion, uterine perforation, and IUD expulsion.

In our analysis, 5.46% of the first IUD insertion attempts (5.64% of all attempts) were unsuccessful. Insertion failure rates vary significantly across studies from as low as<1% in a study that analyzed data from 22 international sites [7] to as high as 17.8% in a U.S.-based prospective study evaluating copper IUD insertion by nurse practitioners [8]. In a comparative study of the safety of IUD insertion by physicians and nurses in Brazil [9], 4.6% of 860 insertion attempts were unsuccessful. In a UK-based retrospective study [10], failure to insert IUD was 8.8%, and insertions by less experienced providers had a significantly higher risk of failure. Thus, the IUD insertion failure rate found in our study is well within the reported range.

The perforation rate of 0.27% (or 2.7/1000) in our analysis is also within the literature-reported range of 1.9 to 3.6 per 1000 insertions [11], [12]. Our findings of higher perforation rate among breastfeeding women, particularly those within the first 3-month postpartum, are consistent with the European Active Surveillance Study (EURAS) IUD study [13], in which the copper IUD perforation rate was 1.1/1000 insertions overall, but 3.7/1000 in breastfeeding women; the EURAS IUD secondary analysis looking at complete perforations [14] identified breastfeeding and delivery within the last 36 weeks as two factors that independently raised perforation risk (5.6/1000 in breastfeeding women<36-week postpartum compared to 1.7/1000 in breastfeeding women>36-week postpartum).

In contrast, our cumulative IUD expulsion rates of 10.3% at 6 months, 13.6% at 12 months, and 17.0% at 18 months exceed literature-reported rates. Cumulative expulsion rates in a randomized multicenter study of two copper IUDs [15] were 2.4% to 6.0% for the first year of use and 3.4% to 6.7% at 2 years. A multicenter trial of implant and copper IUD users [16] reported cumulative IUD expulsion rates of 9.4% at 12 months and 13.1% at 24 months. A prospective study in the UK [17] found cumulative copper IUD expulsion rates of 6% at 12 months and up to 13% at 60 months. Secondary analysis of the Contraceptive CHOICE Project [18] reported cumulative copper IUD expulsion rates of 5.7% at 12 months, 8.6% at 24 months, and 10.7% at 36 months and, similar to our findings, higher rates among nulliparous women (6.0% at 12 months, 10.3% at 24 months, and 14.3% at 36 months) compared with parous women (5.5%, 7.5%, and 8.2% respectively). Although, in both CHOICE and our study, the increased risk among nulliparous women did not reach statistical significance.

Possible explanations for the higher IUD expulsion rates in our study include a lack of clinical experience among newly trained ECHO clinicians. This may also explain expulsion rates being as high as 21.2/100 person-years during the first 3 month following training (Table 5). Additionally, it is possible that expulsions were undercounted in some studies that did not specify if their expulsion rates include both complete and partial expulsions [15], [16] or relied on self-reporting [18]. Another factor may be the frequency of ECHO follow-up visits, which included scheduled quarterly visits, interim visits when participants had any concerns, and protocol-mandated pelvic exams at month 1 and final visits. Coupled with an overall retention of 93.6% across all follow-up visits in the trial, these frequent contacts with trial clinicians helped to identify many partial expulsions that may have been missed in women who receive the IUD from regular family planning providers or in studies with lower retention, less frequent follow-up, fewer pelvic exams, and/or follow-up based on self-report.

A strength of our study is that participants were randomly assigned to their contraceptive methods. This minimized potential self-selection bias and allowed for the assessment of skill-based outcomes of IUD insertion in women with different demographic characteristics. Another strength is our large sample size, high retention rate, and prospective follow-up with frequent visits assessing IUD-related problems and presence, allowing for accurate detection of partial and complete expulsion.

However, because the ECHO case report forms were designed to primarily measure HIV acquisition risk, as opposed to assessing providers’ IUD skills, we were not able to link outcomes to the type of provider, certain provider characteristics, or position of the uterus. For the same reason, we can not know which insertion attempts following initial insertion failure were done, as we recommended, by a backup gynecologist and which by ECHO clinicians. But, even if we conservatively assume that all subsequent failed attempts to place the IUD belonged to ECHO clinicians and all successful ones to a backup gynecologist and exclude the latter from the analysis set, the overall insertion failure rate for ECHO clinicians would remain under 6% and within literature-reported range. Additionally, our findings should be generalized with caution, because in our study, newly trained clinicians received continuous clinical support and refresher training based on needs identified during the quality improvement visits. Also, ECHO clinicians had continuous opportunities to master their new clinical skills. On the contrary, in traditional family planning settings, ongoing support after initial training is often lacking and IUD clients may be few and far between, making it difficult for providers to gain confidence and maintain/refine new skills.

In conclusion, skill-based clinical outcomes in our study, such as IUD insertion failure and uterine perforation, were comparable to the ones reported in the literature, and IUD expulsion rates decreased after the initial 3 month as clinicians gained experience, suggesting clinicians with no prior experience in IUD insertion can provide IUDs safely when they receive competency-based training, ongoing clinical support, and continuous opportunities to apply new clinical skills.

## ECHO Trial Consortium

Jared M. Baeten (University of Washington, Seattle, WA, USA), James Kiarie (WHO, Geneva, Switzerland), Timothy D. Mastro (FHI 360, Durham, NC, USA), Nelly R Mugo (Kenya Medical Research Institute, Nairobi, Kenya & University of Washington, Seattle, WA, USA), Helen Rees (Wits Reproductive Health and HIV Institute, Johannesburg, South Africa).

## Study site principal investigators

Eswatini, Manzini: Jessica Justman, Zelda Nhlabatsi (Family Life Association of Eswatini & ICAP at Columbia University, New York, NY, USA). Kenya, Kisumu: Elizabeth A. Bukusi, Maricianah Onono (Kenya Medical Research Institute, Nairobi, Kenya). South Africa, Brits: Cheryl Louw (Madibeng Centre for Research). South Africa, Cape Town: Linda-Gail Bekker, Gonasagrie Nair (University of Cape Town & Desmond Tutu HIV Centre). South Africa, Durban: Mags Beksinska, Jennifer Smit (MatCH Research Unit (MRU), Faculty of Health Sciences, University of the Witwatersrand). South Africa, East London: G. Justus Hofmeyr, Mandisa Singata-Madliki (University of Fort Hare & University of the Witwatersrand). South Africa, Edendale: Jennifer Smit (MatCH Research Unit [MRU], Faculty of Health Sciences, University of the Witwatersrand). South Africa, Johannesburg: Thesla Palanee-Phillips (Wits Reproductive Health and HIV Institute, Faculty of Health Sciences, University of the Witwatersrand). South Africa, Klerksdorp: Raesibe Agnes Pearl Selepe (The Aurum Institute). South Africa, Ladysmith: Sydney Sibiya (Qhakaza Mbokodo Research Clinic). South Africa, Soshanguve: Khatija Ahmed (Setshaba Research Centre). Zambia, Lusaka: Margaret Phiri Kasaro, Jeffrey Stringer (UNC Global Projects Zambia & University of North Carolina at Chapel Hill, Chapel Hill, NC, USA).

## Other members of the ECHO Trial Consortium

Deborah Baron (Wits Reproductive Health and HIV Institute, Faculty of Health Sciences, University of the Witwatersrand, Johannesburg, South Africa), Deborah Donnell (University of Washington and Fred Hutchinson Cancer Research Center, Seattle, WA, USA), Peter B Gichangi (International Centre for Reproductive Health–Kenya & Technical University of Mombasa, Mombasa, Kenya), Kate B. Heller (University of Washington, Seattle, WA, USA), Nomthandazo Mbandazayo (Wits Reproductive Health and HIV Institute, Johannesburg, South Africa), Charles S. Morrison (FHI 360, Durham, NC, USA), Kavita Nanda (FHI 360, Durham, NC, USA), Melanie Pleaner (Wits Reproductive Health and HIV Institute, Faculty of Health Sciences, University of the Witwatersrand, Johannesburg, South Africa), Caitlin W. Scoville (University of Washington, Seattle, WA, USA), Kathleen Shears (FHI 360, Washington, DC, USA), Petrus S. Steyn (WHO, Geneva, Switzerland), Douglas Taylor (FHI 360, Durham, NC, USA), Katherine K. Thomas (University of Washington, Seattle, WA, USA), Julia D. Welch (FHI 360, Durham, NC, USA), Irina Yacobson (FHI 360, Durham, USA).

## Author contributions

I.Y. wrote the initial draft. V.W. analyzed the data. K.K.T. contributed to methodology and data analysis. C.W.S. performed data curation. K.N. contributed to results interpretation. K.N., M.M., M.P., N.R.M., and I.Y. provided initial training or subsequent clinical support for study clinicians, or both. All authors (I.Y., K.N., V.W., J.K., T.P.P., K.A., T.C., N.R.M., C.L., M.M., M.P., P.G., C.W.S., S.M., K.K.T.) contributed to the execution of the trial, critically reviewed all drafts, and approved the finalized manuscript. The content is solely the responsibility of the authors and does not necessarily represent the views, decisions, or policies of the institutions with which they are affiliated, the ECHO Trial funders, or the supporting governments.

## Footnotes

1 The checklist adapted from the Training Resource Package for Family Planning. United States Agency for International Development (USAID)/ World Health Organization (WHO)/ United Nations Population Fund (UNFPA). https://www.fptraining.org/training/copper-intrauterine-devices-iuds/downloads# (last accessed on March 9, 2023).

## References

[1] United Nations, Department of Economic and Social Affairs, Population Division. Trends in Contraceptive Use Worldwide 2015 (ST/ESA/SER.A/349); 2015.

[2] Buhling K.J., Zite N.B., Lotke P., Black K., INTRA Writing Group (2014). Worldwide use of intrauterine contraception: a review. Contraception.

[3] Rowlands S., Oloto E., Horwell D.H. (2016). Intrauterine devices and risk of uterine perforation: current perspectives. Open Access J Contracept.

[4] Heinemann K., Reed S., Moehner S., Minh T.D. (2015). Risk of uterine perforation with levonorgestrel-releasing and copper intrauterine devices in the European Active Surveillance Study on Intrauterine Devices. Contraception.

[5] Gehani M., Pal M., Arya A., Singh S., Kaushik S., O’Connell K. (2019). Could EAISI-trained providers provide better quality of IUD services? Results of a secondary data analysis of complications as a proxy indicator. Gates Open Res.

[6] Evidence for Contraceptive Options and HIV Outcomes (ECHO) Trial Consortium (2019). HIV incidence among women using intramuscular depot medroxyprogesterone acetate, a copper intrauterine device, or a levonorgestrel implant for contraception: a randomised, multicentre, open-label trial. Lancet.

[7] Chi I.C., Rogers S. (1983). Failure to insert an intrauterine device. Contracept Deliv Syst.

[8] Dermish A.I., Turok D.K., Jacobson J.C., Flores M.E., McFadden M., Burke K. (2013). Failed I.U.D. insertions in community practice: an under-recognized problem?. Contraception.

[9] Lassner K.J., Chen C.H., Kropsch L.A., Oberle M.W., Lopes I.M., Morris L. (1995). Comparative study of safety and efficacy of IUD insertions by physicians and nursing personnel in Brazil. Bull Pan Am Health Organ.

[10] Farmer M., Webb A. (2003). Intrauterine device insertion-related complications: can they be predicted?. J Fam Plann Reprod Health Care.

[11] Chi I., Feldblum P.J., Rogers S.M. (1984). IUD--related uterine perforation: an epidemiologic analysis of a rare event using an international dataset. Contracept Deliv Syst.

[12] Kaneshiro B., Aeby T. (2010). Long-term safety, efficacy, and patient acceptability of the intrauterine Copper T-380A contraceptive device. Int J Womens Health.

[13] Heinemann K., Reed S., Moehner S., Minh T.D. (2015). Risk of uterine perforation with levonorgestrel-releasing and copper intrauterine devices in the European Active Surveillance Study on Intrauterine Devices. Contraception.

[14] Heinemann K., Barnett C., Reed S., Möhner S., Do Minh T. (2017). IUD use among parous women and risk of uterine perforation: a secondary analysis. Contraception.

[15] (1994). A randomized multicentre trial of the Multiload 375 and TCu380A IUDs in parous women: three-year results. UNDP/UNFPA/WHO/World Bank, Special Programme of Research, Development and Research Training in Human Reproduction: IUD Research Group. Contraception.

[16] Bahamondes L., Brache V., Meirik O., Ali M., Habib N., Landoulsi S. (2015). WHO Study Group on Contraceptive Implants for Women. A 3-year multicentre randomized controlled trial of etonogestrel- and levonorgestrel-releasing contraceptive implants, with non-randomized matched copper-intrauterine device controls. Hum Reprod.

[17] Cox M., Tripp J., Blacksell S. (2002). UK Family Planning and Reproductive Health Research Network. Clinical performance of the Nova T380 intrauterine device in routine use by the UK Family Planning and Reproductive Health Research Network: 5-year report. J Fam Plann Reprod Health Care.

[18] Madden T., McNicholas C., Zhao Q., Secura G.M., Eisenberg D.L., Peipert J.F. (2014). Association of age and parity with intrauterine device expulsion. Obstet Gynecol.

